# Stable composition of gut microbiome in the Asian ladybeetle *Coccinella septempunctata* reared on natural and artificial diets

**DOI:** 10.1038/s41598-023-49885-6

**Published:** 2024-01-02

**Authors:** Qiu-Cheng Lu, Jia-Min Yu, Hong-Ling Liu, Xing-Long Wu, Shu-Jun Wei, Min Lei, Peng Cai, Heng-Guo He, De-Qiang Pu

**Affiliations:** 1 Institute of Plant Protection, Sichuan Academy of Agricultural Sciences, Chengdu, 610066 China; 2 China West Normal University, Nanchong, 637002 China; 3 Sichuan Tobacco Company, Chengdu, 653100 China; 4 Institute of Plant and Environmental Protection, Beijing Academy of Agriculture and Forestry Sciences, Beijing, 100097 China; 5 Horticultural Institute, Sichuan Academy of Agricultural Sciences, Vegetable Germplasm Innovation and Variety Improvement Key Laboratory of Sichuan Province, Chengdu, 610066 China

**Keywords:** Entomology, Microbial ecology

## Abstract

The Asian ladybeetle, *Coccinella septempunctata*, is an important insect of predatory natural enemy, which has a strong control effect and application prospects for aphids, whiteflies, mealybugs, and other small-sized pests of agriculture and forestry crops. Gut microbiota composition posed impacts on development of insects. In order to clarify the effect of artificial feed feeding on the intestinal microbial species and structure of *C. septempunctata*, we compared the intestinal microbial flora of *C. septempunctata* reared on bean aphids and artificial food for 15 days. Results show that Proteobacteria was the dominant component in all groups at phylum level, *Rhodobacter*, *Methylovigula, Burkholderia*, and *Bradyrhizobium* were the dominant bacteria among all groups at genus level. As to the differences in bacterial community structure and diversity, there is no significant difference between Shannon index and Simpson index, the principal components analysis of the bacterial communities, and the samples were roughly distributed in different regions. After 15 days of feeding, artificial diet did not significantly reduce the microbial diversity of the gut of *C. septempunctata* compared to the aphid group, and there was no significant effect on the abundance of dominant flora in the gut of *C. septempunctata*, *C. septempunctata* gut has a similar core microbiota. This study clarifies the effects in intestinal microbial diversity and composition structure of the *C. septempunctata* with artificial diet, and provides a theoretical basis for understanding the intestinal microorganisms and optimizating the artificial diet of *C. septempunctata*.

## Introduction

The Asian ladybeetle, *Coccinella septempunctata* (Linnaeus) (Coleoptera: Coccinellidae), is widely distributed in all provinces and regions of China, which is an important insect of predatory natural enemy in agriculture^1^. *C. septempunctataa* is a generalist predator that prey on aphids, whiteflies, thrips, and scale insects. Aphids (Hemiptera: Aphididae) are among the most destructive and abundant pests of agricultural crops worldwide^2^. Pea aphid *Aphis craccivora* (Hemiptera: Aphididae) is one of the key pests of pulse crops worldwide, which has a broad host range, infesting crops such as faba bean, lupine, alfalfa, lentil, chickpea, grass pea and pea^3^, is a typical prey of *C. septempunctata*^4^, which constantly secrete a large amount of nectar by feeding on plant juices to meet their own needs. In this process, pea aphid can also use plant viruses as vectors to spread their own viruses in an effective manner, making them as one of the most destructive pests^5^ .

*C. septempunctata* is a polyphagous insect, which is a natural enemy for comprehensive pest control due to its wide variety of food^6^. At present, *C. septempunctata* is fed on aphids that are reared at large scales. This conventional feeding method is expensive and labor-intensive^7^. Studies on artificial diet for ladybeetles started in the 1950s and continue to date^8^. In 1958, the first formulated artificial diet was successfully used to raise a variety of predatory lady beetles with dry prey powder as the main component^9^. Subsequently, research on the artificial diet of lady beetles shifted from prey components to non-prey components, mestic and foreign biocontrol scholars have studied many kinds of artificial feeds for predatory ladybirds^10–12^. With artificial feeds being used to multiply predator numbers, biological control is becoming more efficient. However, the amount and the quality of food have a direct impact on the growth and reproduction of insects^13^, therefore, determining the effects of different diets on the development and population maintenance of *C. septempunctata* is a crucial part of mass production and biocontrol.

In recent years, attention has been focused on the intestinal microbiota. During the long history of evolution, a reciprocal relationship exists between insects and their gut microbes, and this interaction undoubtedly drives insect evolution^14^. Firstly, insect provides the foundation for gut microbes' growth, providing the necessary habitats and nutrition, in the other hand, the gut intestinal microbiota also has ramifications for the host. Gut microbiota not only promotes digestion and absorption of food by the host^15^, and it also plays a significant role in host development^16^, immunity^17^, aging^18^, and defense against pathogen invasion^19^.

Numerous studies have suggested that diet affects the composition and structure of gut microbiota. Food plays a crucial role in regulating the composition and structure of gut microbiota, both short- and long-term effects are evident^20^. In hosts with flexible diets, such as humans and mice, diet has the potential to alter gut microbiomes over a short period of time^21^. In insects, herbivorous, arboreal species of ants harbour a greater abundance of bacteria than omnivorous, ground-dwelling species of ants^22^. Insects' gut microbiota was shaped by their diet.

Insects are the world’s most diverse and abundant animals in terms of species diversity and body mass in all ecological habitats^23^. Many studies have shown that intestinal microbes play an important role in insect, intestinal microbes play an important role in insect development^24^. However, only a few studies have examined the gut microbiota of *C. septempunctata* exposed to different diets and most of these studies focused on the efficiency of biological control methods^25–29^.

In this study, we are comparing the composition and structure of the gut microbiota between *C. septempunctata* that were fed on live pea aphids and *C. septempunctata* that were fed on artificial diet. High-throughput sequencing technology was used to sequence the gut microbiota of *C. septempunctata*, and predict the function of the gut microbiome, in order to provide a theoretical basis for further in‐depth research on the gut microbes of ladybeetles, and optimize artificial feed components.

## Results

### Summary of the sequencing data

Deep sequencing of eight gut samples from *C. septempunctata* yielded 209,107 sequences with a total length of 304,078,811 bp and an average length of 1449.35 bp. In total, 21 phyla, 37 classes, 60 orders, 97 families, 137 genera, 145 species, and 6370 OTUs were detected from gut samples at a 97% similarity level (Table 1). Good’s coverage index showed that the estimated values of all samples were over 97%, indicating that all samples reached an appropriate sequencing depth and values could represent the overall structure and composition of the ladybeetles' gut microbiota (Table 2).

**Table 1:** OTU clustering and classification level statistics in the gut of adult *Coccinella septempunctata*.

Sample	Reads num	OUT num	Classification					
			Phylum	Class	Order	Family	Genus	Species
ArM	16,943	1404	8	12	25	39	59	46
ArF	13,330	1946	8	12	25	33	48	32
ApF	16,239	1421	9	14	26	46	62	48
ApM	14,299	2175	12	14	28	41	54	40
All	60,811	6370	21	37	60	97	137	145

OTU (Operational Taxonomic Units) is a set of identical markers artificially assigned to a taxonomic unit (strain, genus, species, grouping, etc.) for the purpose of analysis in phylogenetic or population genetics research. In order to obtain the species classification information corresponding to each OTU, the uclust algorithm was used to perform taxonomic analysis on the representative sequences of OTUs, and the community composition of each sample was statistically analyzed at each classification level.

*ArM* male adults fed with artificial diet for 15 days, *ArF* female adults fed with artificial diet for 15 days, *ApM* male adults fed with aphids for 15 days, *ApF* female adults fed with aphids for 15 days.

**Table 2:** Alpha diversity index statistics in the gut of adult *Coccinella septempunctata*.

Sample	Coverage	Alpha diversity			
		Ace	Chao	Shannon	Simpson
ArM	0.9738	2019.88 ± 818.77	1870.28 ± 812.11	2.54 ± 0.06	0.46 ± 0.02
ArF	0.9498	2760.49 ± 604.09	2352.20 ± 544.69	3.67 ± 0.28	0.29 ± 0.03
ApF	0.9707	2077.92 ± 714.66	1803.31 ± 624.35	2.97 ± 0.28	0.37 ± 0.02
ApM	0.9472	3060.83 ± 490.91	2686.05 ± 429.84	3.14 ± 0.19	0.43 ± 0.06

The diversity of bacterial communities is reflected through the Shannon and Simpson indices, and the richness of the community is reflected by the Chao1 and ACE indices.

*ArM* male adults fed with artificial diet for 15 days, *ArF* female adults fed with artificial diet for 15 days, *ApM* male adults fed with aphids for 15 days, *ApF* female adults fed with aphids for 15 days.

### Composition in gut microbiota diversity in ladybeetles reared by different diets

The diversity of bacterial communities is reflected through the Shannon and Simpson indices, and the richness of the community is reflected by the Chao1 and ACE indices. Ace and Chao showed that the ACE index and Chao l index of the artificially fed groups were generally higher than those of the aphid group, but the difference was not statistically significant (p > 0.05). A Shannon diversity method and a Simpson diversity method were used to evaluate species diversity. There is no significant difference between the Shannon index and Simpson index (p > 0.05). In addition, in artificial diet group, there was significant difference of Shannon and Simpson indices between female and male ladybirds (p < 0.05), female adults with higher diversity of bacterial communities than males. In aphid group, there was no significant difference in Shannon and Simpson indices between female and male ladybirds (p > 0.05) (Table 2).

### Composition of the gut microbiota in ladybeetles through different feeding methods

**Figure 1:**
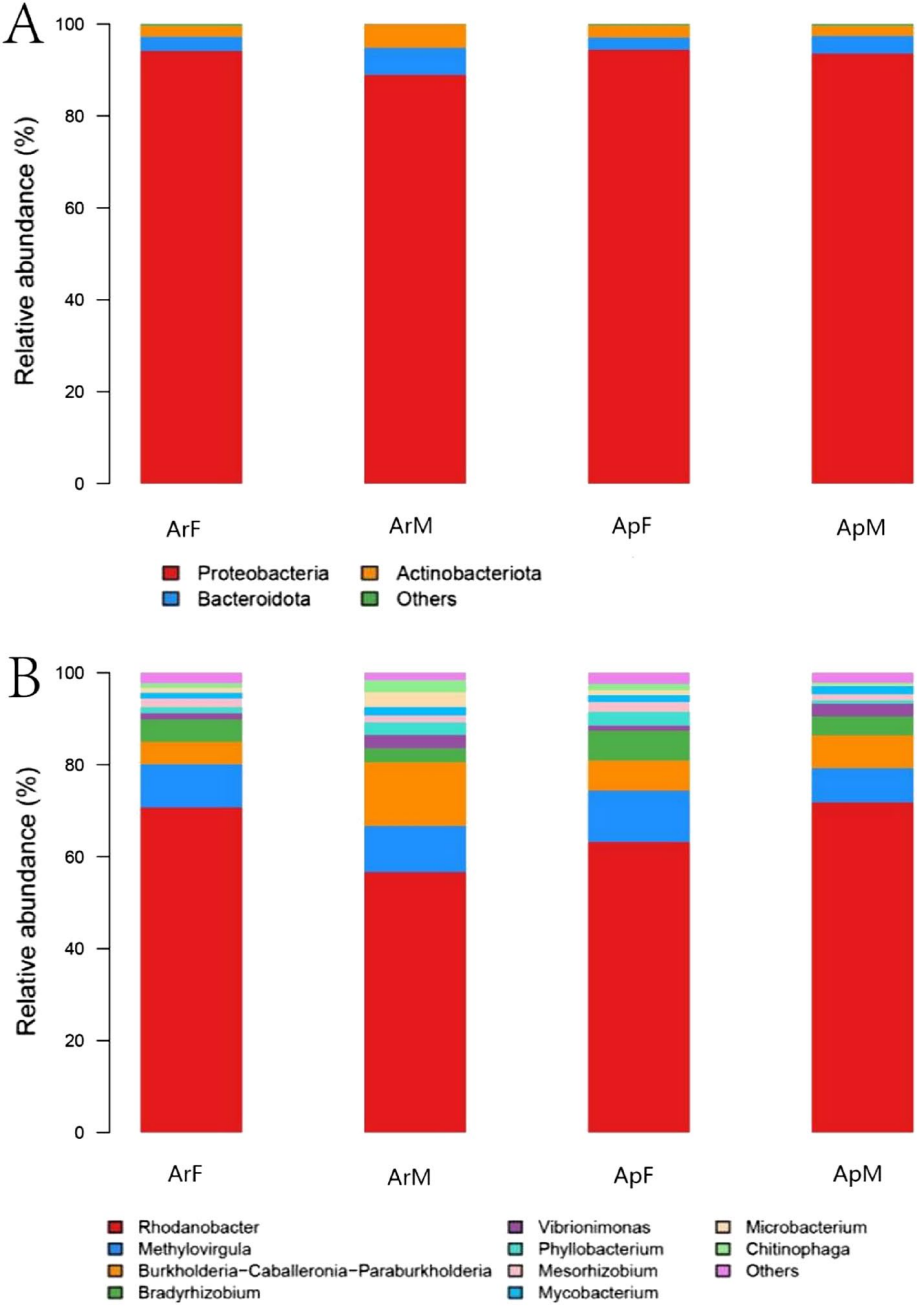
Relative abundance of the dominant phyla (**A**) and genera (**B**) of bacteria in gut of adult *Coccinella septempunctata* in this study. *ArM* male adults fed with artificial diet for 15 days, *ArF* female adults fed with artificial diet for 15 days, *ApM* male adults fed with aphids for 15 days, *ApF* female adults fed with aphids for 15 days. Different colors represent different types of dominant bacterial..

**Figure 2:**
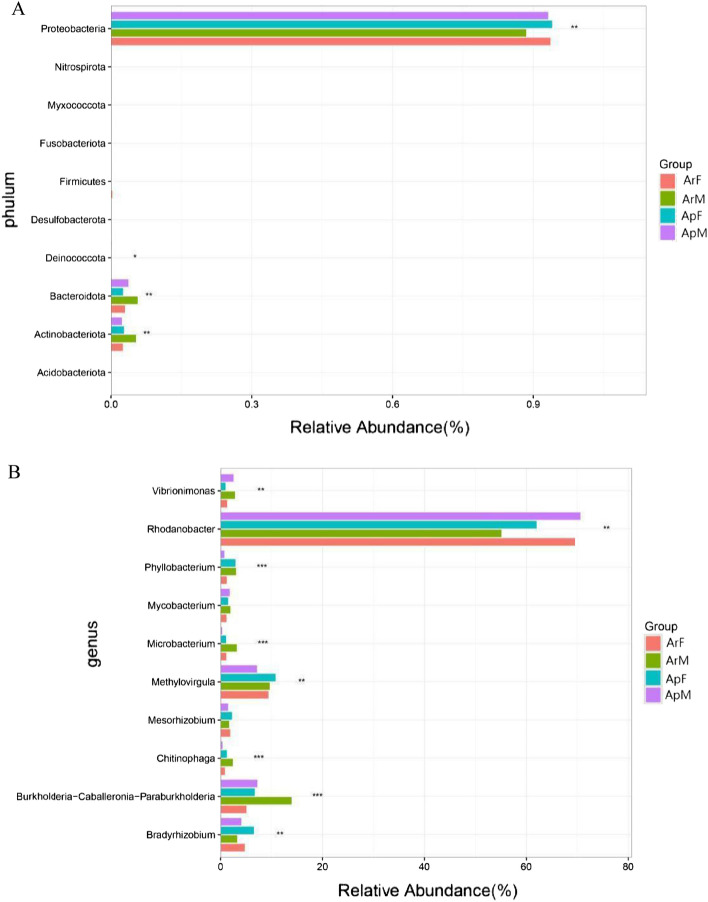
Relative abundance of the top ten phyla (**A**) and genera (**B**) in gut of adult *Coccinella septempunctata*. *ArM* male adults fed with artificial diet for 15 days, *ArF* female adults fed with artificial diet for 15 days, *ApM* male adults fed with aphids for 15 days, *ApF* female adults fed with aphids for 15 days. The vertical axis represents the genus level of classification and the corresponding column length represents the average relative abundance of the gut microbiota species from the four treatment groups. The different colors represent different Groups. On the far right is the value of P, *0.01 < P ≤ 0.05, **0.001 < P ≤ 0.01, ***P ≤ 0.001.

At phylum level of classification, the gut microbiota of ladybeetles consisted mainly of the following bacteria: Proteobacteria, Bacteroidota, Actinobacteriota, Firmicutes, Deinoccota, Myxococcota, Deinoccota, Desulfobacterota, Acidobacteriota, Planctomycetota and Chloroflexi (Fig. 1A). In this study, the top 10 phyla in the guts of ladybeetles from different habitats showed no significant differences in relative abundances. Proteobacteria had the highest relative abundance in ApF (94.12%) and the lowest abundance in ArF (88.63%). Bacteroidetes are influential and consistent component of the ladybeetle's gut, and their relative abundance was highest in ArF (6.02%) and lowest in ApF (2.67%). The relative abundance of actinobacteria was highest in ArF (5.21%) and lowest in ApM (2.39%). In addition, Firmicutes had the highest relative abundance in ArM (0.31%) and the lowest abundance in ArF (0.05%). Furthermore, the relative abundances of the following bacteria were relatively insignificant, not exceeding one percent: Deinococcota, Myxococcota, Deinococca, Acidobacteriota, Planctomycetota and Chloroflexi (Fig. 2B and Table 3).

**Table 3:** Top 10 abundant phyla of bacteria in gut of adult *Coccinella septempunctata*.

Phylum	Different habitats (%)			
	ArM	ArF	ApF	ApM
Proteobacteria	93.80	88.63	94.12	93.19
Bacteroidota	3.12	6.02	2.67	3.95
Actinobacteriota	2.63	5.21	2.84	2.39
Firmicutes	0.31	0.05	0.19	0.13
Deinococcota	0.09	0.05	0.13	0.30
Myxococcota	0.01	0	0.01	0.01
Desulfobacterota	0.01	0.01	0	0.01
Acidobacteriota	0	0	0	0
Planctomycetota	0	0	0	0.01
Chloroflexi	0	0	0	0
Others	0.03	0.03	0.04	0.01

*ArM* male adults fed with artificial diet for 15 days, *ArF* female adults fed with artificial diet for 15 days, *ApM* male adults fed with aphids for 15 days, *ApF* female adults fed with aphids for 15 days.

Results of this study indicated that there were 35 genera with a relative abundance higher than 1% in the gut of ladybeetles (Fig. 1B). The gut microbiota of ladybeetles consisted mainly of the following bacteria: Rhodanobacter, Methylovirgula, Burkholderia-Caballeronia-Paraburkholderia, Bradyrhizobium, Phyllobacterium, Vibrionimonas, Mesorhizobium, Mycobacterium, Microbacterium and Chitinophaga. Within the top 10 gut microbiota of ladybeetles, there were significant differences in the relative abundance of gut microbiota at the genus level of ladybeetles reared on different diets (Table 3). Among them, the genera with the highest relative abundances was *Rhodanobacter*, which was highest (70.73%) in ApM group, and lowest (55.22%) in ArF group. The relative abundance of Methylovirgula was highest (10.92%) in ApF group and lowest (7.25%) in ApM group. Burkholderia-Caballeronia-Paraburkholderia were most abundant (14.02%) in ArF group and least abundant (5.17%) in ArM group and the relative abundance of Bradyrhizobium was highest (6.68%) in ApF group and lowest (3.38%) in ArF group. The relative abundance of Phyllobacterium was highest (3.12%) in ArF group and lowest (0.85%) in ApM group. Vibrionimonas relative abundance was highest (5.08%) in ArM group and lowest (1.07%) in ApF group, Mesorhizobium was most prevalent (2.35%) in ApF group, whilst, the ArM group had the lowest relative abundance (0.09%). The relative abundance of Mycobacterium was highest (2.06%) in ArM group and lowest (1.59%) in ApF group, Microbacterium relative abundance was highest (3.31%) in ArF group and lowest (0.02%) in ArM group, and Chitinophaga relative abundance was highest (2.50%) in ArF group and lowest (0.49%) in ApM group (Fig. 2A and Table 4).

**Table 4:** Top 10 abundant genera of bacteria in gut of adult *Coccinella septempunctata*.

Genera	Different habitats (%)			
	ArM	ArF	ApF	ApM
Rhodanobacter	69.65	55.22	62.12	70.73
Methylovirgula	9.51	9.75	10.92	7.25
Burkholderia-Caballeronia-Paraburkholderia	5.17	14.02	6.84	7.33
Bradyrhizobium	4.86	3.38	6.68	4.20
Phyllobacterium	1.39	3.12	3.01	0.85
Vibrionimonas	5.08	2.94	1.07	2.66
Mesorhizobium	0.09	1.77	2.35	1.58
Mycobacterium	1.93	2.06	1.59	1.91
Microbacterium	0.02	3.31	1.17	0.39
Chitinophaga	1.35	2.50	1.35	0.49
Others	6.08	10.07	8.09	4.21

*ArM* male adults fed with artificial diet for 15 days, *ArF* female adults fed with artificial diet for 15 days, *ApM* male adults fed with aphids for 15 days, *ApF* female adults fed with aphids for 15 days.

### Beta diversity of gut bacteria

**Figure 3:**
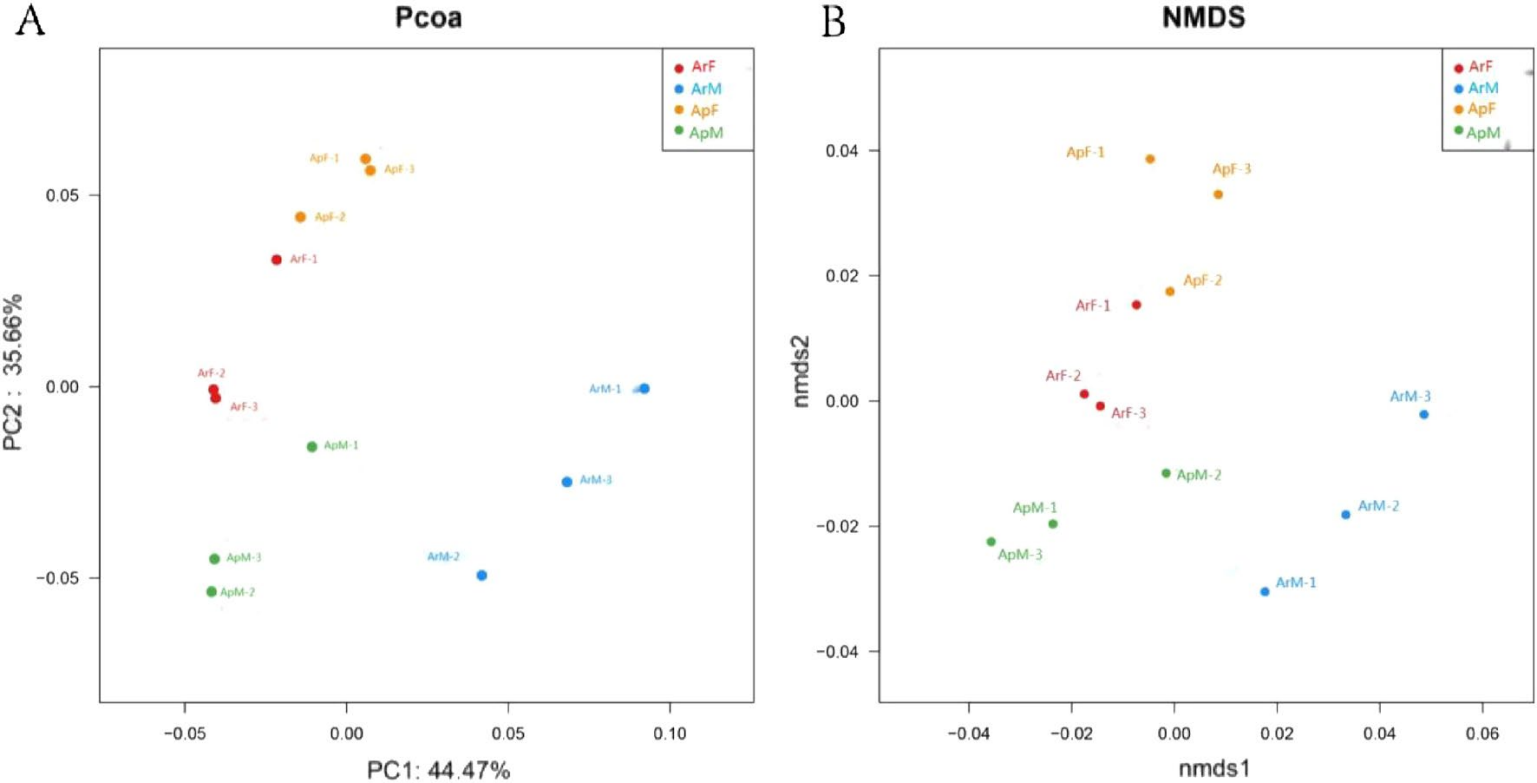
PCA analyses (**A**) and NMDS diagram (**B**) analysis in the intestine of adult *Coccinella septempunctata*. *ArM* male adults fed with artificial diet for 15 days, *ArF* female adults fed with artificial diet for 15 days, *ApM* male adults fed with aphids for 15 days, *ApF* female adults fed with aphids for 15 days. The gut microbiota was analyzed based on the weighted UniFrac distance of a principal coordinates analysis (PCoA) and Bray Curtis distance of the non-metric multidimensional scaling (NMDS) method for different Sample.

The gut microbiota was analyzed based on the weighted UniFrac distance of a principal coordinates analysis (PCoA) and BrayCurtis distance of the non-metric multidimensional scaling (NMDS) method for different sample. PCoA and NMDS analyses revealed that the compositions of the gut microbiota of *C. septempunctata* at different diets were varied from one another (Fig. 3). In PCoA, PC1 accounted for 63.5% of the total variance, and PC2 accounted for 29.18%. Through the PCoA calculated based on the weighted unifrac distance, we can find that each group of *C. septempuncta* samples is relatively discrete. This indicates that the similarity of intestinal microbial diversity among samples in each group is low, and there are differences among individuals (Fig. 3).

**Figure 4:**
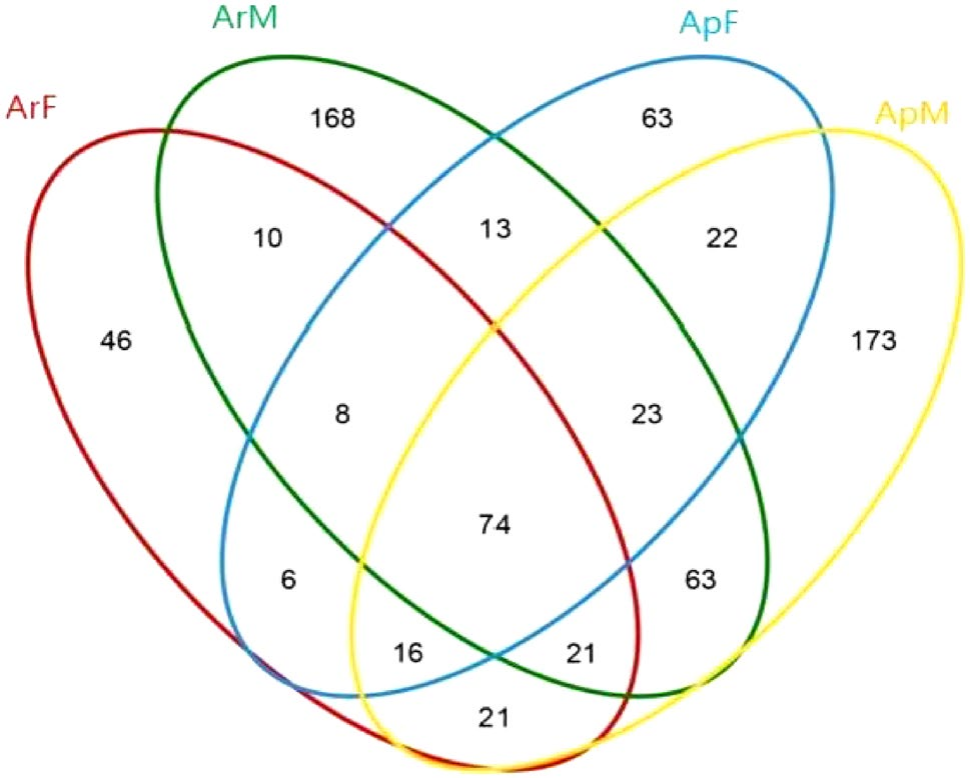
Venn diagram of OTUs of bacteria in the gut of adult *Coccinella septempunctata*. *ArM* male adults fed with an artificial diet for 15 days, *ArF* female adults were fed with artificial diet for 15 days, *ApM* male adults fed with aphids for 15 days, *ApF* female adults were fed with aphids for 15 days. Different colors represent different samples, areas where two circles of different colors overlap represent the number of shared OTUs, and areas where no circles overlap represent the number of unique OTUs.

Venn diagram analysis was used to obtain the overlap of OTUs under different treatments. Results showed that the number of OTUs in artificially fed group was much less than that in aphid group. There were 471 OTUs in artificially fed group, 637 OTUs in aphid group, and 74 OTUs were the same in all groups. This indicated that the bacterial community composition of the intestines of the artificially fed group and the aphid group differed greatly, and the aphid group was richer in flora. In addition, under the same feeding conditions, there were greatly differences in the composition of gut microbiota between females and males (Fig. 4).

### Functional analysis of gut microbiota

In order to compare the potentially functional capacity of different feeding gut microbial communities of ladybeetles, the abundance of different sample flora at the level of the KEGG primary and secondary pathways was predicted using PICRUSt (Table 2). All annotated to KEGG first-level pathway: metabolism (69.82%), genetic information processing (12.51%), environmental information processing (10.35%), cellular processes (0.45%), genetic information processing (14.06%), human diseases (0.16%) and organismal systems (0.12%), respectively. The abundance of metabolic functional pathways accounted for more than half of the abundance of all primary pathways, therefore this shows that the gut bacteria of *C. septempunctata* mainly perform the function of metabolizing various substances (Table 5).

**Table 5:** KEGG level 1 pathway abundance of bacteria in the gut of adult *Coccinella septempunctata*.

Pathway name	ArM	ArF	ApF	ApM
Metabolism	21,313,853	18,199,839	21,270,829	18,023,851
Genetic information processing	3,806,576	3,020,611	3,649,855	3,202,181
Environmental information Processing	3,203,499	2,907,971	3,320,681	2,711,319
Cellular processes	1,406,282	1,132,220	1,379,373	1,196,700
Organismal systems	389,121	324,413	380,687	331,329
Human diseases	510,615	455,972	532,221	426,250

The abundance of different sample flora at the level of the KEGG primary pathways was predicted using PICRUSt. All annotated to KEGG first-level pathway.

*ArM* male adults fed with artificial diet for 15 days, *ArF* female adults fed with artificial diet for 15 days, *ApM* male adults fed with aphids for 15 days, *ApF* female adults fed with aphids for 15 days.

A total of 11 secondary KEGG pathways were identified by mapping and annotating the secondary pathways in the metabolic pathways of the adult intestinal flora (Table 3). The corresponding functional names of these secondary pathways and their percentage of the total sample were: carbohydrate metabolism (22.55%), amino acid metabolism (21.32%), energy metabolism (13.42%), metabolism of cofactors and vitamins (9.07%), nucleotide metabolism (7.91%), biodegradation and metabolism of xenobiotics (6.50%), lipid metabolism ( 6.60%), metabolism of other amino acids (5.34%), metabolism of terpenoids and polyketides (3.21%), and Biosynthesis and metabolism of sugars (2.97%), and biosynthesis of other secondary metabolites (2.05%). This indicates that the adult gut flora of *C. septempunctata* are mainly involved in the metabolism of carbohydrates and amino acids, while a small proportion is involved in the metabolism of sugars, terpenoids, and polyketides. The abundance of these functions showed significant differences, consistent with the level 1 KEGG pathways (Table 6).

**Table 6:** KEGG pathway (metabolism pathway) abundance of bacteria in the gut of adult *Coccinella septempunctata*.

Pathway name	ArM	ArF	ApF	ApM
Carbohydrate metabolism	4,533,895	3,923,237	4,549,245	3,823,709
Biosynthesis of other secondary metabolites	437,249	363,536	425,913	372,277
Xenobiotics biodegradation and metabolism	1,467,149	1,404,482	1,580,870	1,256,733
Energy metabolism	2,863,891	2,342,822	2,817,641	2,394,541
Lipid metabolism	1,407,662	1,247,730	1,429,264	1,197,205
Nucleotide metabolism	1,687,921	1,362,463	1,635,365	1,421,351
Amino acid metabolism	4,523,006	3,881,668	4,501,857	3,842,628
Metabolism of other amino acids	1,139,079	963,649	1,130,662	968,481
Glycan biosynthesis and metabolism	633,354	507,886	600,090	539,422
Metabolism of cofactors and vitamins	1,934,564	1,609,146	1,910,542	1,623,616
Metabolism of terpenoids and polyketides	686,083	593,221	689,380	583,888

The abundance of different sample flora at the level of the KEGG secondary pathways was predicted using PICRUSt. All annotated to KEGG level 2 pathway (metabolism pathway).

*ArM* male adults fed with artificial diet for 15 days, *ArF* female adults fed with artificial diet for 15 days, *ApM* male adults fed with aphids for 15 days, *ApF* female adults fed with aphids for 15 days.

## Discussion

*C. septenuctata* is an important predatory insect that preys on aphids, whiteflies, mites and eggs/young larvae of lepidopterans, and its gut microbiota plays an instrumental role in its growth and reproduction^1,30,31^. Studies have shown that when there is a decline in the amount of beneficial flora, this increases the chance of viral infections and ultimately decreases the survival rate of the host^32^. Gut microbiota is of critical importance for the rearing of *C.* *septempunctata*. Microbes were significantly correlated with host development, aging, and social behavior^33,34^. Sex, feeding, different developmental stages, and adaptation to the environment of insects are all factors that affect the diversity and composition of gut microorganisms in insects^35–37^. This study found that artificial feed affected the composition and structure of the gut microbiota of *C.* *septempunctata*, but there was no significant difference in its diversity. In addition, many studies have shown that diet is one of the most significant variables influencing the gut microbial community^38–40^. Insects gut microbiota composition is affected by different diets and the type and number of gut microbes can change, as well as the dominant flora^41^. Despite this, not much research has been conducted on *C. septempunctata*'s gut microbes using various diets. Despite this, there are not many studies on the intestinal microbial diversity of the ladybug under different feeding conditions, which is not conducive to the protection and cultivation of the ladybug. Therefore, this study used high-throughput sequencing technology to explore the gut microbes of *C. septempunctata* on different diets.

In this study, based on the high-throughput sequencing technology of 16S rDNA, the gut bacterial community diversity and divergence of two adult populations of aphid-feeding *C. septempunctata* and artificially fed *C. septempunctata* were analysised. In total there were 26 phyla, 64 classes, 154 orders, 247 families and 433 genera. Alpha diversity analysis revealed that both the diversity and richness of gut bacterial communities were higher in aphid-feeding *C. septempunctata* compared to artificially fed *C. septempunctata*. Microbiota can influence nutrient extraction from dietary sources, and diet can also impact gut microbiota composition and function at the same time^42^. Therefore, it is speculated that the reason for this may be that aphids are natural prey of insects and therefore the nutrients obtained from this source are more suitable for growth and development in ladybeetles. Research show that aphids, such as *A. pisum*, can be handled and consumed more easily by predators, thus making them as a more suitable prey in terms of net energy gain^43^. This was further supported by results obtained in PCA analysis of two populations, which exhibited significant differences in the composition and structure of the microbial community.

The diversity of gut microorganisms varies between insects of different sexes^44^. The alpha diversity analysis in this experiment showed that the diversity of intestinal bacteria in female *C. septempunctata* was higher than that in male *C. septempunctata* in the artificial diet group, indicating that the diversity of intestinal bacteria of *C. septempunctata* is also affected by gender differences. The same phenomenon has also been found, in intestines of male and female silkworms, the abundance of 23 bacterial genera in male is more than 1.5 times that of female, while the abundance of 7 bacterial genera in female is more than 1.5 times that of male^45^. There are also significant differences in the diversity of gut microorganisms between male and female adults of *Chrysoperla sinica*^46^. Gender differences may also lead to different risks of autoimmune (type I) diabetes in female and male mice^47^. However, in aphid group, alpha diversity analysis showed no significant difference in gut microbial diversity between male and female ladybirds. Therefore, we speculate that it may be due to aphids fed have more nutrients suitable for the growth and development of ladybirds, and the food plays a major role in the changes of the gut microbial community in the ladybirds.

This experiment found that Proteobacteria was the most prevalent bacteria in both the aphid diet-fed and artificial diet-fed feeding groups, which was found throughout nature, including in animals and plants^48^. Proteobacteria is associated with diverse metabolisms such as decomposition, fermentation of complex sugars, and the production of vitamins and degraded aromatic compounds, boosting their hosts' absorption of nutrients^49^. Previous study show that Proteobacteria dominate the gut microbiome of Gypsy moths^50^, Bombyx^51^and cotton bollworms^52^, similar findings also have been reported in many other species of insects in the family Cerambycidae^53,54^. The aforementioned studies suggested that Proteobacteria are widely found in gut microbiota of insects and the dominant role of Proteobacteria in the insect gut microbiota may be a distinctive feature of insect gut microbiota composition. In addition, in comparison with previous studies on the intestinal flora of *C. septempunctata*, we found that the intestinal core flora of *C. septempunctata* is mainly composed of Proteobacteria, Firmicutes, and Bacteroides^53^. Another study reported the species of Cerambycidae adult gut bacteria of Korea and found the dominant phyla were Proteobacteria, Firmicutes and Bacteroidetes^55^. It has been found that Firmicutes have the ability to encode enzymes for energy metabolism, biosynthesize vitamin B, and can produce enzymes for digestion and absorption of various substances, thereforehelping their hosts to digest and absorb nutrients^56^. Microbes of the Bacteroidetes are primarily responsible for decomposing carbohydrates through carbohydrate-active enzymes^57^, Microbes of the Bacteroidetes are primarily responsible for decomposing carbohydrates through carbohydrate-active enzymes^57^.

At the genus level, we discovered that Rhodanobacter was the most dominant bacterial genera in ArM, ArF, ApF and ApM groups. Among them, the ApM group had the highest abundance. Studies have revealed that Rhodanobacter genera can utilize various carbon sources, including cellobiose^58^. In larvae of longhorned beetles that feed on plants rich in carbohydrates (cellulose and hemicellulose) and lignin, Rhodanobacter can help the larvae digest more carbon nutrients through carbon sequestration^54^. We speculate that the highest abundance of Rhodanobacter bacteria in the ApM group may come from the aphid fed diet, which contains many sources of carbon. Thus, the function of *Rhodanobacter* genera may be associated with nutrient metabolism in the insect gut. Furthermore, Methylovirgula, Burkholderia- Caballeronia-Paraburkholderia, Mesorhizobium and Bradyrhizobium are also the dominant genera of bacteria in each group. As far as we know, Methylovirgula has not been identified in *C. septempunctata* and its biological function is unknown. Methylovirgula is ubiquitous in soil and has been found in many soil samples as a major species producing carbon activity, scholars have found that the microorganism has the highest content in mixed peat swamp forest systems and has the effect of harnessing and reducing methane^59^. Burkholderia, Mesorhizobium and Bradyrhizobium are commonly found in plant roots and they all have nitrogen fixation abilities. Burkholderia belongs to the class of β-proteobacteria and is a bona fide genus of archaebacteria that separates itself from group II of the Pseudomonas rRNA gene^60^. The genus was originally proposed by Yabuuchi and over 80 species of Burkholderia have been reported so far. A Burkholderia bacterium is known for its ability to fix nitrogen, promote plant growth, and degrade recalcitrant carbohydrates and aromatic compounds like L-phenylalanine^61^. Strains of Burkholderia have been reported in citrus root samples, and evidence suggests that these strains may be able to trigger the expression of genes associated with disease resistance^62,63^. However, the biological effects of Burkholderia on the *C. septempunctata* is unclear, and it is speculated that it may participate in the metabolism of carbohydrates. Rhizobia, a group of soil bacteria with which legume plants interact symbiotically, forms nitrogen-fixing root nodules on the roots of legume plants as a result of their symbiotic interactions^4^. It is important to note that Mesorhizobium and Bradyrhizobium are also two other types of nodules, these nodules fix the diazine in the atmosphere into ammonia, which is used as a nitrogen source by the host plant^64^. As far as this genus of bacteria is concerned, most of what we know about them revolves around their capacity to fix nitrogen in plant roots, and their role on insects remains unclear. We speculate that the presence of Mesorhizobium and Bradyrhizobium in ladybird beetles may come from their aphid food source, in particular when the aphids feed on broad beans.

Insects digest and absorb food in their guts, and intestinal flora plays an important role in their metabolism and nutrition^65^. In this study, the potential functions of ladybeetle gut bacteria under different food sources were predicted by PICRUSt. In addition, the intestinal flora of *C. septempunctata* mainly metabolizes carbohydrates, amino acids, and other substances to provide adequate nutrition for it. This study also predicted the metabolic functions of gut bacteria for sugars, exogenous chemicals, terpenoids, and polyketides, suggesting that these gut bacteria are involved in the metabolism of sugars, terpenoids, and cellulose, sugar promotes the growth, development, and reproduction of ladybugs^66^. In our research, Serratia was one of the main genera, it has been shown that Serratia are dominant bacteria in the guts of many insects and can help host insects efficiently degrade substances such as cellulose, monoterpenoids or diterpenes^67,68^.

In this study, we completed a comparative analysis of the composition and diversity of adult gut microbes through different feeding in *C. septempunctata* using high-throughput sequencing technology to predict the potential functions of gut microbes. The results showed that after 15 days of artificial feeding, there was no significant change in the gut microbial diversity and dominant bacterial species of the *C. septempunctata. C. septempunctata* gut has a similar core microbiota, the dominant phyla at the level of the intestinal flora are Proteobacteria, Firmicutes and Bacteroidetes, and the dominant genera are Rhodanobacter, Bradyrhizobium, Ralstonia, Asinibacterium and Burkholderia. By PICRUSt prediction analysis, the gut microorganisms of *C. septempunctata* in this study mainly functioned to metabolize various substances, mainly carbohydrates and amino acids, and to a lesser extent sugars, terpenoids and polyketides. However, more studies were needed to further analyze the interaction between host and microbiota in different rations, as well as the structure and function of microorganisms. Follow-up studies will be conducted to screen and validate the intestinal tract with these bacteria. This will be done to further clarify the functions of the relevant intestinal microbes and their mechanisms, in order to provide theoretical basis and ideas for the subsequent in-depth study on the gut microorganisms of heptastar and optimization of artificial feed components.

## Methods

### Insect sampling

Both aphids and *C. septempunctata* were obtained from the insect laboratory at Institute of Plant Protection, Sichuan Academy of Agricultural Sciences, China. The main components of the artificial diet were dried bee pupae powder, sucrose, dried pig liver powder, rape pollen, pea aphid powder, β-carotene (2% active ingredient) and dried banana powder. In this study, two diet regimes were used and a total of four groups were divided by distinguished between male and female and feeding time. (1) Females were fed with an artificial diet for 15 days (ArF), (2) Males were fed with an artificial diet for 15 days (ArM), (3) Males were fed with aphids for 15 days (ApM), (4) Females were fed with aphides for 15 days (ApF).

Collect the eggs of *C. septempunctata* reared with aphids, place them in a cage containing live aphids, and until them hatch. The larvae feed on aphids until they enter the adult stage. Then collect the adult worms into a 1000 ml plastic culture box (30 heads per box), a total of 8 groups, for different feeding methods. After feeding for 15 days, immerse the worm body in 70% ethanol for 1 min (pipette 1 ml into a 1.5 ml sterile centrifuge tube), repeatedly invade it 3 times, and then rinse it with sterile water 5 times to remove the surface DNA of the worm. Then write the name of the sample on the sterile tube or centrifuge tube, record all sample information, and place the collection tube in liquid nitrogen for temporary storage; Finally, it is stored in a dry ice box and sent to the sequencing company. Groups ApM and ApF were fed ~ 150–170 aphid heads per day to prevent *C. septempunctata* from feeding on each other. The artificial fed groups, ArF and ArM, were fed with both dry powder and 20% sucrose liquid (sucrose: purified water = 2:8) supplied separately. Place five grams of dry powder in a plastic sheet, place the sucrose solution on the cotton wolf, roll it into a ball, place it in the bottle cap, and then put it together in a feeding box. The food was chaged daily using steritle equipment. Visually observe and record every 24 h. All insects were kept indoors under the following environmental conditions: temperature (25 ± 1) °C, relative humidity 75% ± 7%, photoperiod 10 L:14 D, light intensity 18,000 LX.

### DNA extraction and PCR amplification

Microbial DNA was extracted from *C. septempunctata* samples using the E.Z.N.A.® Soil DNA Kit (Omega Bio-tek, Norcross, GA, U.S.) according to manufacturer’s protocols. The V1-V9 region of the bacteria 16S ribosomal RNA gene were amplified by PCR (95 °C for 2 min, followed by 27 cycles at 95 °C for 30 s, 55 °C for 30 s, and 72 °C for 60 s and a final extension at 72 °C for 5 min. The primers primers 27F 5′-AGRGTTYGATYMTGGCTCAG-3′ and 1492R 5′-RGYTACCTTGTTACGACTT-3′, were used which included an eight-base barcode sequence unique to each sample. PCR reactions were performed in triplicate using a 20 μL mixture containing 4 μL of 5 × FastPfu Buffer, 2 μL of 2.5 mM dNTPs, 0.8 μL of each primer (5 μM), 0.4 μL of FastPfu Polymerase, and 10 ng of template DNA. Amplicons were extracted from 2% agarose gels and purified using the AxyPrep DNA Gel Extraction Kit (Axygen Biosciences, Union City, CA, U.S.) according to the manufacturer’s instructions.

### Library construction and sequencing

SMRTbell libraries were prepared from the amplified DNA by blunt-ligation according to the manufacturer’s instructions (Pacific Biosciences). Purified SMRTbell libraries from Zymo and HMP mock communities were sequenced on dedicated PacBio Sequel II 8 M cells using the Sequencing Kit 2.0 chemistry. Purified SMRTbell libraries from the pooled and barcoded samples were sequenced on a single PacBio Sequel II cell. All amplicon sequencing was performed by Shanghai Biozeron Biotechnology Co. Ltd (Shanghai, China).

### Processing of sequencing data

PacBio raw reads were processed using the SMRT Link Analysis software version 9.0 to obtain demultiplexed circular consensus sequence (CCS) reads with the following settings: minimum number of passes = 3, minimum predicted accuracy = 0.99. Raw reads were processed through SMRT Portal to filter sequences for length (< 800 or > 2500 bp) and quality.Sequences were further filtered by removing barcodes, primer sequences, chirmas and sequences if they contained 10 consecutive identical bases.

OTUs were clustered with 98.65% similarity cutoff using UPARSE (version 7.1 http://drive5.com/uparse/) and chimeric sequences were identified and removed using UCHIME. The phylogenetic affiliation of each 16S rRNA gene sequence was analyzed by RDP Classifier (http://rdp.cme.msu.edu/) against the silva (SSU132) 16S rRNA database using confidence threshold of 70%^69^.

### Alpha- and beta-diversity analyses

The rarefaction analysis based on Mothur v.1.21.1 was conducted to reveal the diversity indices, including the Chao, ACE, and Shannon diversity indices. The beta diversity analysis was performed using UniFrac^60^ to compare the results of the principal component analysis (PCA) using the community ecology package, R-forge (Vegan 2.0 package was used to generate a PCA figure). Mantel tests were carried out to examine the Spearman’s rank correlation between the factor and the bacterial community similarity using Bray–Curtis distance matrices with 999 permutations, using the vegan package in R. Multivariate analysis of variance (MANOVA) was conducted to further confirm the observed differences. The Spearman’s correlation coefficients were assessed to determine the relationships between microbiota and chemical factors such as signaling molecules. Correlation was considered significant when the absolute value of Spearman’s rank correlation coefficient (Spearman’s r) was > 0.6 and statistically significant (P < 0.05). All statistic analysis were performed by R stats package. The R (pheatmap package) and Cytoscape (http://www.cytoscape.org) were applied to visualize the relationships through correlation heatmap and network diagrams, respectively. Redundancy analysis (RDA) was employed to explore the relationship between environmental factors and bacterial communities. One-way analysis of variance (ANOVA) tests were performed to assess the statistically significant difference of diversity indices between samples. Differences were considered significant at p < 0.05. Venn diagrams were drawn using online tool “Draw Venn Diagram” (http://bioinformatics.psb.ugent.be/webtools/Venn) to analyze overlapped and unique OTUs during the treatment processes. One-way permutational analysis of variance (PERMANOVA) was performed using R vegan package to assess the statistically significant effects of treatment processes on bacterial communities.

### Functional prediction of the microbial genes

Phylogenetic Investigation of Communities by Reconstruction of Unobserved States (PICRUSt) (http://picrust.github.io/picrust/tutorials/genome_prediction.html) program based on the Kyoto Encyclopedia of Genes and Genomes (KEGG) database was used to predict the functional alteration of microbiota in different samples. The OTU data obtained were used to generate BIOM files formatted as input for PICRUSt v1.1.09 with the make.biom script usable in the Mothur. OTU abundances were mapped to Greengenes OTU IDs as input to speculate about the functional alteration of microbiota (Supplementary Tables S1–S3).

### Supplementary Information

Supplementary Table 1.

Supplementary Table 2.

Supplementary Table 3.

## Data Availability

The datasets generated and/or analysed during the current study are available in PRJNA963975 repository, https://www.ncbi.nlm.nih.gov/bioproject/PRJNA963975.
